# Inhibition of tau aggregation by the CCT3 and CCT7 apical domains

**DOI:** 10.1002/pro.70162

**Published:** 2025-05-22

**Authors:** Miki Ben‐Maimon, Nadav Elad, Segev Naveh‐Tassa, Yaakov Levy, Amnon Horovitz

**Affiliations:** ^1^ Department of Chemical and Structural Biology Weizmann Institute of Science Rehovot Israel; ^2^ Department of Chemical Research Support Weizmann Institute of Science Rehovot Israel

**Keywords:** Alzheimer's disease, CCT/TRiC, chaperonins, protein aggregation, tau

## Abstract

The eukaryotic chaperonin containing t‐complex polypeptide 1 (CCT/TRiC) is a molecular chaperone that assists protein folding in an ATP‐driven manner. It consists of two stacked identical rings that are each made up of eight distinct subunits. Here, we show that the apical domains of subunits CCT3 and CCT7 from humans are strong inhibitors of tau aggregation, which is associated with several neurological disorders such as Alzheimer's and Parkinson's diseases. Kinetic analyses and negative‐stain electron microscopy indicate that the mechanism of inhibition of tau aggregation by the apical domains of subunits CCT3 and CCT7 differ. Aggregation of tau alone, or in the presence of the apical domain of subunit CCT7, can be described by a fragmentation model whereas in the presence of the apical domain of subunit CCT3, it fits a saturating elongation and fragmentation mechanism. Coarse‐grained molecular dynamics simulations show that tau interacts with different regions in the apical domains of subunits CCT3 and CCT7, in agreement with their different inhibition mechanisms.

## 1 INTRODUCTION

Protein misfolding and aggregation are toxic to cells. Consequently, mechanisms for the prevention of protein aggregation and rescuing or degrading misfolded proteins are found in all kingdoms of life. Such mechanisms include protein degradation and molecular chaperone machineries (Balchin et al. 2016; Hartl and Hayer‐Hartl 2009). A ubiquitous family of molecular chaperones is the chaperonins, which includes the eukaryotic chaperonin containing t‐complex polypeptide 1 (CCT/TRiC) (Grantham 2020; Horovitz et al. 2022; Lopez et al. 2015; Smith and Willardson 2022; Willison 2018). CCT/TRiC assists protein folding in an ATP‐fuelled manner and is essential for the correct folding of certain proteins, including some involved in critical cellular functions, such as actin and tubulin (Sternlicht et al. 1993). It consists of two stacked oligomeric rings that form a cavity in which folding can take place. Each ring consists of eight distinct subunits (CCT1, CCT2, CCT3, CCT4, CCT5, CCT6, CCT7, and CCT8) (Kalisman et al. 2013; Leitner et al. 2012). These subunits share a similar architecture and consist of an apical domain responsible for substrate binding, an equatorial domain housing an ATP‐binding site, and an intermediate domain that connects the apical and equatorial domains and facilitates intra‐ring allosteric communication (Kalisman et al. 2013; Leitner et al. 2012).

The hetero‐oligomeric structure of CCT/TRiC may have evolved, in part, owing to the selective advantage provided by the acquisition of subunit‐specific functions either within the intact complex or outside of it. An example for the latter case is the function of CCT2 in promoting the autophagic degradation of solid protein aggregates (Ma et al. 2022; Roy et al. 2023). Other examples for functions outside the complex include the role of CCT5 as a component of the serum response factor signaling pathway through its interaction with the co‐transcriptional activator MRTF‐A (Elliott et al. 2015) and the role of CCT4 as a binding partner of the dynactin complex protein p150Glued (Echbarthi et al. 2018; Spiess et al. 2015). In other cases, free subunits can possess functions related to their role in the intact complex. For example, the apical domain of subunit CCT1 (apiCCT1) was found to inhibit polyglutamine aggregation (Tam et al. 2006). Other findings suggested that subunit CCT3, in the intact complex or free, also interacts with polyglutamine (Nadler‐Holly et al. 2012; Zhao et al. 2016). CCT3 in the intact complex (as well as CCT6) was also found to interact with α‐synuclein amyloid fibrils (Sot et al. 2017). By contrast, subunit CCT7 or its apical domain was never reported to have an independent function outside the complex. Here, we show that human apiCCT7 and apiCCT3 both have a strong inhibitory effect on tau aggregation.

Tau is an intrinsically disordered protein that plays a crucial role in stabilizing microtubules in neuronal cells, ensuring proper cellular function and axonal transport (Fung et al. 2020; Kadavath et al. 2015). Though it is natively unfolded in solution, post‐translational modifications, truncations, or mutations can promote the formation of toxic oligomers and fibrils, which are linked to neurological disorders such as Alzheimer's and Parkinson's diseases (Alonso et al. 2001; Despres et al. 2017; Mandelkow and Mandelkow 2012; Ramesh et al. 2020). Tau consists of an N‐terminal projection domain, four microtubule‐binding repeat domains, and a C‐terminal tail. In the human central nervous system, it exists in six isoforms that vary in the presence or absence of the second microtubule‐binding repeat domain and/or two inserts in the N‐terminal projection domain (Gustke et al. 1994).

In this study, we utilized the tau^4R^ construct (also named K18), which is comprised of the four microtubule‐binding repeat domains (Barghorn et al. 2004). This construct aggregates relatively rapidly in vitro and has, therefore, been a widely used model for investigating tau aggregation (Baggett and Nath 2022; Irwin et al. 2021; Ramachandran and Udgaonkar 2012). Tau fibrillation involves several microscopic steps that include primary nucleation, fibril growth through monomer addition and rearrangement, and fibril fragmentation (Yao et al. 2020). Various molecular chaperones, such as Hsp70, ATP‐independent small heat shock proteins, DNAJA2, DNAJB1, and Hspb1, have been shown to inhibit fibril formation and facilitate the degradation of misfolded tau species (Caballero et al. 2021; Dou et al. 2003; Irwin et al. 2021; Mok et al. 2018; Petrucelli et al. 2004; Ryder et al. 2022; Voss et al. 2012). Different chaperones can affect distinct steps in tau aggregation. For example, HSPB1 primarily interacts with the tau^4R^ monomer, thereby preventing nucleation, whereas chaperones from the Hsp40 family inhibit aggregation mainly by interacting with its seeds and fibrils (Irwin et al. 2021). Here, we show that tau^4R^ aggregation can also be inhibited by the apical domains of human CCT3 and CCT7 subunits. Interactions between tau and CCT subunits within or outside of the CCT complex have not yet been reported. Our findings are, however, in line with previous work that showed lower expression of CCT subunits in Alzheimer's disease patients and with age (Brehme et al. 2014).

## 2 RESULTS AND DISCUSSION

Kinetic analysis of protein aggregation necessitates a high level of reproducibility, which can be challenging to achieve, as noted before in the case of monitoring tau^4R^ fibril formation via the change in thioflavin T (ThT) fluorescence (Ramachandran and Udgaonkar 2012). We found that reproducibility can be attained by adding a urea wash to the tau^4R^ purification process to remove DNA contamination, as well as by storing tau^4R^ immediately after gel filtration without prior concentration to avoid dimer formation. Reproducibility was also found to be enhanced by carrying out the aggregation assays in the presence of a high concentration of dithiothreitol (DTT) to maintain reducing conditions and by agitating the samples using orbital shaking, as previously described for α‐synuclein aggregation (Giehm and Otzen 2010). Using these procedures, we were able to obtain very reproducible aggregation curves for tau^4R^ alone (Figure S1, Supporting Information) but reproducibility is decreased when aggregation is slowed down (e.g., at low concentrations of tau^4R^ or in the presence of inhibitors).

**FIGURE 1:**
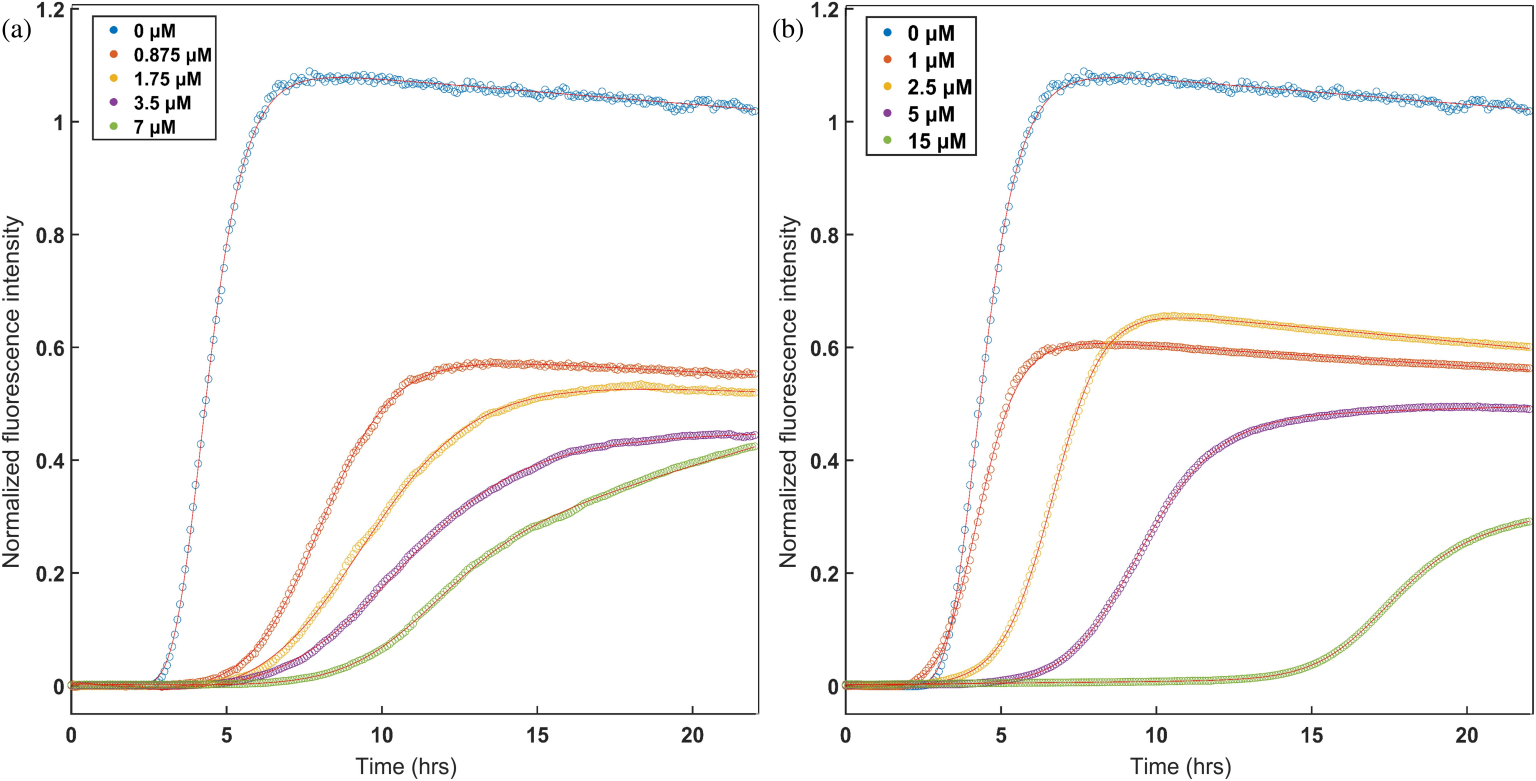
Inhibition of tau^4R^ fibril formation by the CCT3 and CCT7 apical domains is dose dependent. The kinetics of tau^4R^ fibril formation were monitored in the presence of different concentrations of the apical domains of CCT3 (a) and CCT7 (b) by measuring ThT fluorescence as a function of time as described in section 3. At least 12 curves were collected for each condition in two or three independent experiments and averaged. The continuous red lines through the data points are fits to Equation (1).

Having established conditions for reproducible tau^4R^ aggregation assays, we proceeded to test the effects of individual CCT apical domains on tau^4R^ aggregation kinetics. The apical domains of all eight human CCT subunits were expressed, but only apiCCT3, apiCCT7, and apiCCT8 could be highly purified in soluble monomeric form (Figure S2). Work on the other apical domains was, therefore, not continued. Strikingly, both apiCCT3 and apiCCT7 were found to have a significant, dose‐dependent effect on tau^4R^ aggregation (Figures 1 and S3). Fits of the aggregation curves to Equation (1) show, for example, that the aggregation half‐time of tau^4R^ increases from 4.41 ± 0.02 h to 11.22 ± 0.05 h in the presence of 7 μM apiCCT3 and to 17.60 ± 0.16 h in the presence of 15 μM apiCCT7. The half‐time of tau^4R^ aggregation alone is significantly different from those of the aggregation in the presence of apiCCT3/7, as the probability that they are derived from the same distribution has a vanishingly small *p*‐value (*Z* scores >25). The apparent aggregation rate constant also changed. It decreases from 1.79 ± 0.04 h in the case of tau^4R^ alone to 0.69 ± 0.01 h in the presence of 7 μM apiCCT3 and 0.72 ± 0.02 h in the presence of 15 μM apiCCT7. ApiCCT8 also exhibited a mild inhibitory effect on tau^4R^ aggregation, but it was not quantified due to variability between replicates, whose cause remains unclear.

**FIGURE 2:**
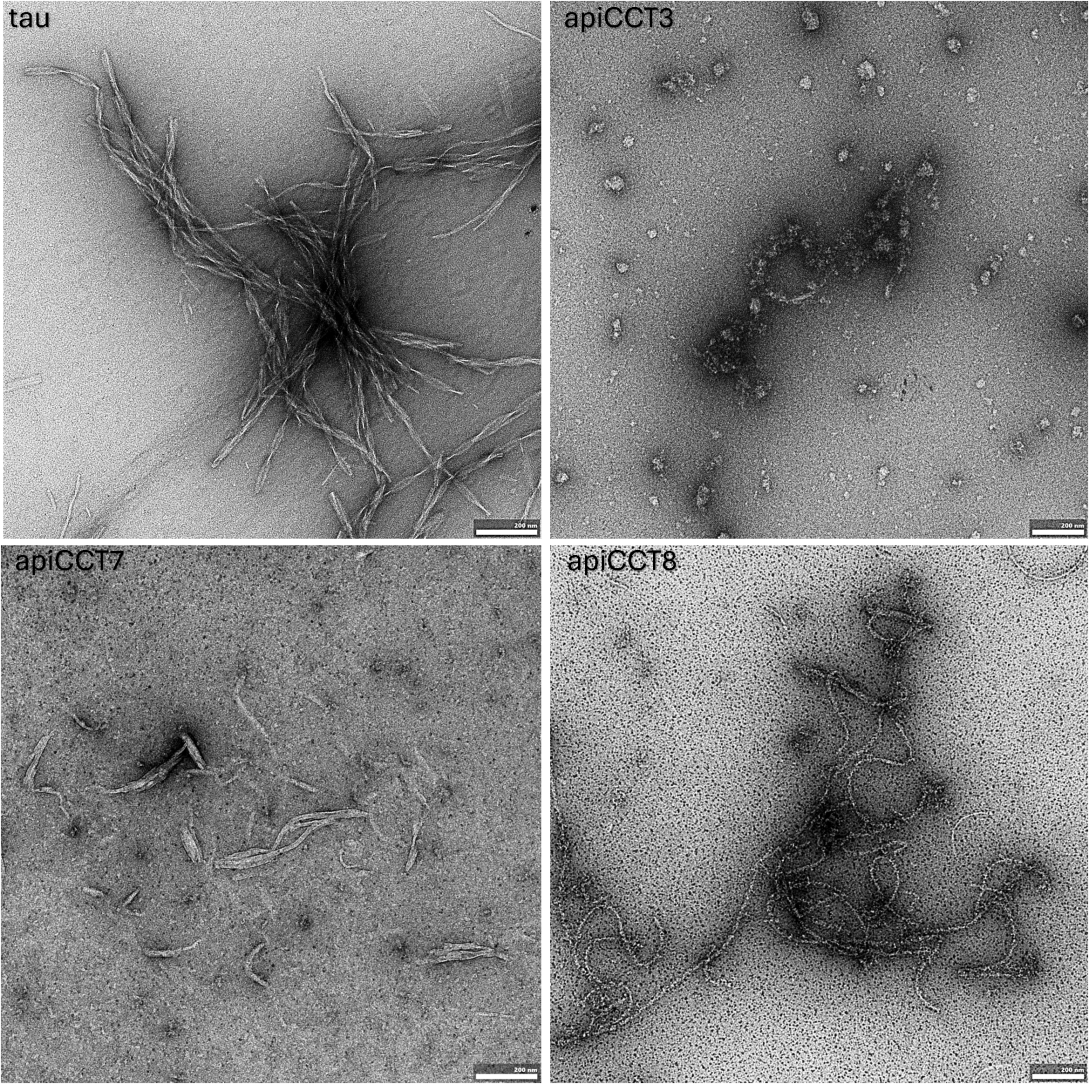
Negative stain electron microscopy of tau^4R^ fibrils. Representative images show tau^4R^ fibrils alone and the effect of adding 15 μM of apiCCT3, apiCCT7, and apiCCT8. Samples were prepared and imaged as described in section 3. The scale bars correspond to 200 nm.

The effects on tau^4R^ aggregation of apiCCT3, apiCCT7, and apiCCT8 were also characterized using negative‐stain transmission electron microscopy (TEM) (Figure 2). Aggregates of tau^4R^ alone were found to form paired‐helical filaments as reported before (Kidd 1963). In the presence of apiCCT3, no filaments were detected, whereas only short filaments were found in the presence of apiCCT7. These results agree with the observed ThT fluorescence after 23 h being about 30% (Figure 1) and 2.5% (not shown) in the presence of apiCCT7 and apiCCT3, respectively, relative to tau^4R^ alone. Interestingly, C‐shaped filaments were observed in the presence of apiCCT8, a morphology previously reported for a fragment of tau^4R^ comprising residues 242–364 with the P301L/V337M double mutation (Frost et al. 2008). The TEM imaging is consistent with the kinetic data, but the distinct morphologies observed in the presence of the different apical domains suggest that their aggregation inhibition mechanisms may differ.

**FIGURE 3:**
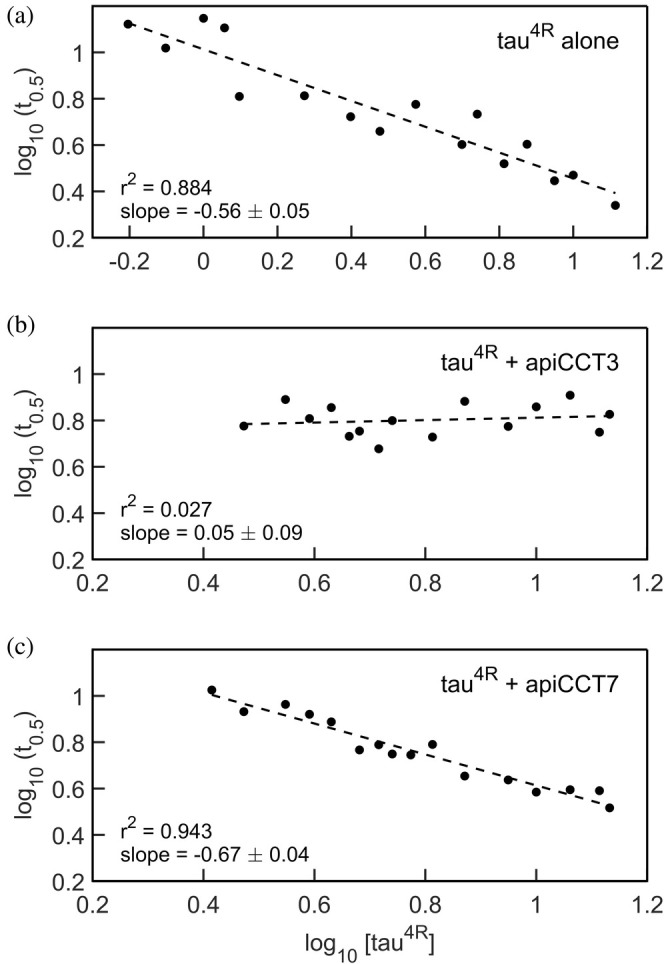
Double‐logarithmic plots of aggregation half‐time as a function of tau^4R^ concentration in the absence or in the presence of apiCCT3 or apiCCT7. Aggregation half‐times (t_0.5_, hours) were estimated from fits to Equation (1) of plots of ThT fluorescence as a function of time at varying tau^4R^ concentrations (in μM) alone (a) or in the presence of 1.75 μM apiCCT3 (b) or 2.5 μM apiCCT7 (c), as described in section 3. The dashed lines represent the linear fits to the data. The concentration of apiCCT3 was selected to be lower than that of apiCCT7 as a smaller amount of the former was sufficient to induce a significant change in t_0.5_. The errors in the values of log_10_(t_0.5_) are under 3%.

Insight into aggregation mechanisms can be obtained by measuring the aggregation half‐time at different monomer concentrations of the aggregating protein, that is, tau^4R^. Early work suggested that double‐logarithmic plots of aggregation lag‐time versus monomer concentration should be linear with a slope corresponding to half the number of monomers forming the nucleus (Oosawa and Asakura 1975). More recent work has shown, however, that double‐logarithmic plots of aggregation half‐time versus monomer concentration can be nonlinear or linear depending on the aggregation mechanism (Meisl et al. 2016). In the linear case, the relationship between the slope and nucleus size also depends on the aggregation mechanism. Here, linear relationships are observed (Figure 3), which are consistent with nucleation elongation, secondary nucleation, or fragmentation mechanisms but rule out other mostly more complex ones such as nucleation elongation combined with fragmentation (Meisl et al. 2016). In the case of tau^4R^ alone, a slope of −0.56 ± 0.05 is observed, which is like the slope of −0.65 obtained for a tau construct comprising residues 304–380 (Rodriguez Camargo et al. 2021). In the presence of apiCCT7, a slope equal to −0.67 ± 0.04 is observed, which is similar to that observed in the case of tau^4R^ alone. By contrast, the value of the slope, in the presence of apiCCT3, is 0.05 ± 0.09, thereby providing further evidence that the aggregation inhibition mechanisms of apiCCT3 and apiCCT7 differ.

**FIGURE 4:**
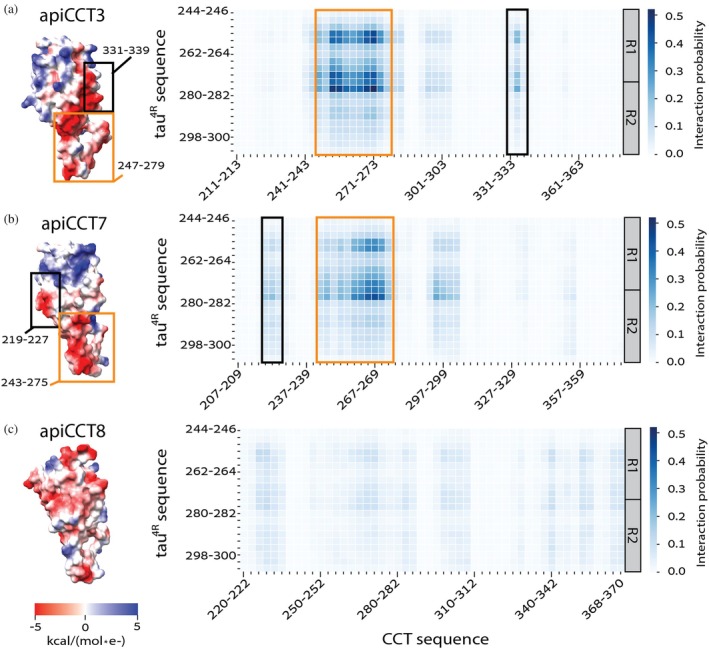
Structural analysis of the interaction between tau^4R^ and CCT apical domains. Heat maps show interaction probabilities between tau residues and residues in apiCCT3 (a), apiCCT7 (b), and apiCCT8 (c), which range from 0 (white, no interaction) to 0.5 (dark blue, high interaction probability). The heat maps were generated for the interactions of groups of three consecutive residues in tau^4R^ (y‐axis) with groups of three consecutive residues in the CCT apical domains (x‐axis). Only the sequence corresponding to the R1 and R2 regions of tau^4R^ is shown since the interactions of the apical domains with R3 and R4 are minimal. The electrostatic potential of each apiCCT is shown on the left using a color code of red to blue for surface potential from −5 to 5 in units of kcal/mol*e^−^, respectively. The black boxes in the structures and corresponding heat maps indicate regions that differ between apiCCT3 and apiCCT7 in their interaction with tau^4R^ whereas the orange boxes indicate the region that interacts with tau^4R^ in both domains.

The data collected for different concentrations of tau^4R^ either alone or in the presence of apiCCT7 were subjected to global fitting using AmyloFit (Meisl et al. 2016) (Figure S4). AmyloFit can be used to analyze kinetic data of aggregation acquired in the presence of different concentrations of the aggregating species to determine the aggregation mechanism. Given the linear correlations we found here (Figure 3), only the mechanisms for which no curvature is expected, that is, nucleation elongation, secondary nucleation, and fragmentation, were considered. In the case of tau^4R^ alone or in the presence of apiCCT7, the best fits, as indicated by the lowest mean residual errors, are obtained for the fragmentation model. The values of the slopes in Figure 3 for tau^4R^ alone and in the presence of apiCCT7 are also most consistent with the fragmentation model. Hence, our data indicate that apiCCT7 inhibits tau^4R^ aggregation without changing its mechanism. AmyloFit could not be used to fit the data in the presence of apiCCT3 since there is little change in the aggregation half‐time. The close‐to‐zero (0.05 ± 0.09) slope of the double‐logarithmic plots of aggregation half‐time versus monomer concentration may indicate that the aggregation mechanism when apiCCT3 is present is saturating elongation and fragmentation (Meisl et al. 2016). The fragmentation model consists of one elongation step whereas the saturating elongation and fragmentation model involves two elongation steps. Hence, it is possible that apiCCT7 acts like a competitive inhibitor of elongation whereas apiCCT3 is a non‐competitive inhibitor, that is, it dissociates from a tau monomer only after it binds the growing fibril. The different aggregation mechanisms of tau^4R^, in the presence of apiCCT3 or apiCCT7, are consistent with the results of coarse‐grained molecular dynamics simulations. The simulation data show that both apical domains interact with tau^4R^ via residues 245–272 but that in the case of apiCCT3 also residues 331–339 are involved whereas the interaction of apiCCT7 is mediated also by residues 219–227 (Figure 4). The simulations also show that the interaction of apiCCT8 with tau^4R^ is very weak, in agreement with its weak inhibitory effect.

In summary, our finding that tau^4R^ aggregation is inhibited by apiCCT3 and apiCCT7 suggests that these proteins may have therapeutic potential that needs to be explored further. An initial evaluation of such potential will be to test for inhibition of tau aggregation by apiCCT3 and apiCCT7 in neuron‐like cells. Guided by the simulations, another future line of exploration will be to identify experimentally, for example, by mutagenesis, the regions in apiCCT3/7 that interact with tau^4R^. These results may enable the design of smaller but still active versions of apiCCT3/7 that may have greater therapeutic potential. Our results suggest that a function of the full CCT/TRiC complex and/or individual CCT subunits may be to inhibit tau aggregation in vivo. Such a biological role of CCT/TRiC is consistent with the involvement of both CCT subunits (Chen 2019) and tau (Wang and Mandelkow 2016) in axonal transport. It is also consistent with the association of tau with various neurodegenerative diseases (Wang and Mandelkow 2016) and increasing evidence that impaired function of CCT/TRiC is associated with a range of brain disorders (Kraft et al. 2024).

## 3 MATERIALS AND METHODS

### 3.1 Expression and purification of CCT3/7/8 apical domains

The gene for the human CCT3 apical domain (residues 223–383) fused to a C‐terminal His_6_‐tag was amplified with the respective forward and reverse primers: 5′‐ATTTTGTTTAACTTTAAGAAGGAGATATACATATGGACTCCTGTGTCTTGCGTG‐3′ and 5′‐CTGCCTTGCGGCCGCTTAGTGGTGGTGGTGGTGGTGGCTAGCCCCCCGGAGGAGAAT‐3′. The gene for the human CCT7 apical domain (residues 207–376) fused to a C‐terminal His_6_‐tag was amplified using the respective forward and reverse primers: 5′‐ATTTTGTTTAACTTTAAGAAGGAGATATACATATGGATTCTCAGCTGGTAGCTGGTG‐3′ and 5′‐CTGCCTTGCGGCCGCTTAGTGGTGGTGGTGGTGGTGGCCACGGAGAATGAAGGTGCAT‐3′.

The gene for the human CCT8 apical domain (residues 220–369) fused to a C‐terminal His_6_‐tag was amplified using the respective forward and reverse primers: 5′‐ATTTTGTTTAACTTTAAGAAGGAGATATACATATGGGCATGGTTTTTAAGAAGGA‐3′ and 5′‐CTGCCTTGCGGCCGCTTAGTGGTGGTGGTGGTGGTGATCTTCCTTTTCATGCTTAAA‐3′. The amplified sequences were digested with NotI and NdeI and cloned into the pET27b plasmid which had been digested with the same enzymes.

*Escherichia coli* BL21 (DE3) cells harboring these plasmids were grown overnight at 37°C in LB medium containing 0.03 mg/mL kanamycin. The cultures were diluted 1:100, grown until an OD_600_ of 0.8 was reached and expression was then induced by adding 0.5 mM isopropyl‐β‐D‐thiogalactoside (IPTG). Growth was continued overnight at 18°C and the cells were then centrifuged (7650*g*, 60 min) and stored at −80°C. The cell pellets were resuspended in 40 mM Tris–HCl (pH 8.0) buffer containing 5 mM imidazole, 0.5M NaCl, and 5 mM β‐mercaptoethanol (buffer A) to which 20 μL EDTA‐free protease inhibitor cocktail (Calbiochem) and 0.0016 mg/mL DNase were added. Cell lysis was accomplished using a French press and sonication (20 s, 56 s off, 3 times, 70% amplitude). The lysate was clarified (38,720*g*, 60 min) and loaded (3 mL/min) on a Ni‐NTA affinity column pre‐equilibrated with buffer A. The column was then washed with 25 mL buffer A and elution was carried out using a gradient (16 min, 5 mL/min) of imidazole from 5 to 400 mM in buffer A. Ammonium sulfate was slowly added to the eluted protein to a saturation of 65%, followed by incubation for 1 h at 4°C with stirring. The pellet was collected by centrifugation (30,600*g*, 30 min) and the supernatant was thoroughly removed. The pellet was stored at 4°C for 12–24 h, then resuspended in 0.25 mL (for CCT3) or 1 mL (for CCT7/8) of 20 mM Tris–HCl (pH 8.0) buffer containing 0.5M NaCl, 0.5 mM EDTA, and 1 mM DTT (buffer B), and centrifuged (1700*g*, 5 min) to remove aggregates. In the case of the CCT7/8 apical domains, the samples were further concentrated via centrifugation (4000*g*, 15 min) with an Amicon centrifugal filter unit (Millipore, 10000 MWCO) to a volume of 0.25 mL. The apical domains samples were then injected into a Superdex 75 column pre‐equilibrated with buffer B. Fractions were collected (0.5 mL/min), concentrated via centrifugation (4000*g*, 15 min) with an Amicon centrifugal filter unit (Millipore, 10000 MWCO), and analyzed by SDS‐PAGE. Fractions containing purified CCT3, CCT7, or CCT8 apical domains were combined, and protein concentrations were determined by measuring absorbance at 280 nm using a Nanodrop instrument. The purified proteins were aliquoted, frozen in liquid nitrogen, and stored at −80°C.

### 3.2 Expression and purification of tau^4R^

The gene coding for tau^4R^ (comprising residues 244–372 of the human tau protein) (Barghorn et al. 2004), which contains the mutation C332A and is fused to an N‐terminal His_6_‐tag followed by a tobacco etch virus (TEV) protease cleavage site, was cloned into the pET‐29b(+) plasmid (a gift from R. Rosenzweig, Weizmann Institute) (Irwin et al. 2021).

*Escherichia coli* BL21 (DE3) cells harboring this plasmid were grown overnight at 37°C in LB medium containing 0.03 mg/mL kanamycin. The cultures were diluted 1:100, grown until an OD_600_ of 0.8 was reached, and expression was then induced by adding 1 mM IPTG. Growth was continued overnight at 16°C, and the cells were then centrifuged (7650*g*, 60 min) and stored at −80°C. The cell pellet was resuspended in 50 mM Tris–HCl (pH 8.0) buffer containing 300 mM KCl, 10 mM imidazole, and 5 mM β‐mercaptoethanol (buffer C) to which 20 μL EDTA‐free protease inhibitor cocktail (Calbiochem) and 0.0016 mg/mL DNase were added. Cell lysis was accomplished by sonication (20 s, 56 s off, 6 times, 70% amplitude), followed by heating for 20 min at 95°C and centrifugation (38,720*g*, 60 min). The following steps were carried out at room temperature unless specified otherwise. The supernatant was loaded (2.5 mL/min) on a Ni‐NTA affinity column pre‐equilibrated with buffer C. The column was then washed (5 mL/min) with 25 mL buffer C, 25 mL of buffer C with 6M urea (2.5 mL/min) and again with 25 mL of buffer C. The tau^4R^ protein was eluted (5 mL/min) using a gradient of imidazole from 10 to 300 mM in buffer C. The eluted protein was dialyzed in 25 mM Hepes buffer (pH 8.0) with 300 mM KCl for 2 h at 4°C using SnakeSkin dialysis tubing (ThermoScientific) in the presence of TEV to remove the His_6_‐tag. Following the initial dialysis, the protein was further dialyzed overnight at 4°C in 25 mM Hepes buffer (pH 7.4) containing 1 mM DTT (buffer D).

The cleaved protein was loaded (0.5 mL/min) on a cation‐exchange HiTrap SP FF column that was pre‐equilibrated with buffer D. The column was washed (1 mL/min) with 25 mL buffer D, 25 mL buffer D containing 6M urea, and again with 25 mL buffer D. Tau^4R^ was eluted (1 mL/min) using a gradient of KCl from 0 to 300 mM in buffer D. Fractions containing tau^4R^ were then loaded (5 mL/min) on a Ni‐NTA affinity column pre‐equilibrated with 25 mM Hepes buffer (pH 7.4) containing 300 mM KCl and 1 mM DTT (buffer E). The column was washed with 25 mL buffer E. The tau^4R^ protein was eluted by washing (5 mL/min) the column with buffer E containing 30 mM imidazole. The eluted protein was concentrated to 0.25 mL via centrifugation in an Amicon centrifugal filter unit (Millipore, 10000 MWCO). Fresh DTT (5 mM) was then added, and the protein was injected into a Superdex 75 column pre‐equilibrated with 50 mM Hepes buffer (pH 7.4) containing 50 mM KCl and 2 mM DTT (buffer F). The sample was eluted at 0.5 mL/min. Protein concentration was determined by measuring absorbance at 280 nm using a Nanodrop instrument. The purified protein was aliquoted, frozen in liquid nitrogen, and stored at −80°C. The purity of the proteins was assessed by SDS‐PAGE.

### 3.3 Aggregation assays

The aggregation of different concentrations of tau^4R^ was monitored by measuring the change in fluorescence emission at 485 nm of ThT, upon excitation at 440 nm, as a function of time using a plate reader (TECAN Spark, Tecan Group Ltd., Maennedorf, Switzerland). Tau^4R^ aliquots were thawed at 37°C and then pre‐incubated with 20 mM DTT at 20°C for 20 min to confirm the reduction of disulfide bonds. The reactions were carried out at 20°C in black‐bottom 96‐well plates that were sealed with optical adhesive film to prevent evaporation and subjected to orbital shaking at 300 rpm. Heparin sulfate (Sigma, average MW ~15 kDa) at a 1:1 molar ratio to tau^4R^ was first added to each well followed by buffer B with or without CCT3/7 apical domain. The reactions were initiated by adding a mix containing different concentrations of tau^4R^, 20 mM DTT, and 10 μM ThT. The final buffer composition was 12% buffer B and 88% buffer F. At least 12 curves were collected for each condition in two or three independent experiments and averaged. A control for each treatment in which heparin was absent was carried out, and the resulting curve was subtracted from the average so that the change in fluorescence could be attributed solely to tau^4R^ aggregation.

### 3.4 Analysis of aggregation assays

Each data point, *F*_i_, in the average of the aggregation curves collected in the absence of CCT3/7 apical domains was normalized to *F*_*i*,n_ as follows: *F*_i,n_ = (*F*_i_ − *F*_i,min_)/(*F*_i,max_ − *F*_i,min_), where *F*_i,max_ and *F*_i,min_ designate the initial and maximal values of the curve for tau^4R^ aggregation alone. Aggregation curves collected in the presence of CCT3/7 apical domains were then normalized similarly, using the *F*_i,max_ and *F*_i,min_ values from the averaged curve in their absence.

The midpoint values of the aggregation curves obtained with or without the CCT3/7 apical domain were obtained by fitting the data to an equation describing a sigmoidal transition between two straight lines (Padrick and Miranker 2002):

(1) $$F_{i}\left(t\right)=\frac{m_{1}*t+n_{1}}{1+e^{k*\left(t_{0.5}-t\right)}}+\left(m_{2}*t+n_{2}\right)*\left(1-\frac{1}{1+e^{k*\left(t_{0.5}-t\right)}}\right),$$

where *m*_1_ and *n*_1_ designate the slope and *y*‐intercept, respectively, of the pre‐transition baseline, *m*_2_ and *n*_2_ designate the slope and *y*‐intercept of the post‐transition baseline, *t*_0.5_ corresponds to the midpoint of the transition, and *k* represents the slope of the transition. The fitting was performed using cftool in matlab R2022a.

### 3.5 Negative stain TEM

Carbon‐coated copper grids (electron microscopy sciences) were glow discharged for 90 s at 15 mA. Samples (3 μL) containing the products of the aggregation reactions carried out as described above of 10 μM tau^4R^ alone or in the presence of either 15 μM CCT3, CCT7, or CCT8 apical domains were placed on the grids, incubated for 30 s, and stained with 2% uranyl acetate solution. Imaging was performed on a Tecnai T12 TEM (ThermoFisher Scientific, USA) operated at an accelerating voltage of 120 kV and equipped with a TemCam‐XF416 camera (TVIPS GmbH, Germany).

### 3.6 Coarse‐grained molecular dynamics simulations

Simulations were carried out using an in‐house coarse grained (CG) C_α_‐based native topology model (Bigman and Levy 2020, 2021). Each residue in tau^4R^ and apiCCT3, apiCCT7, or apiCCT8 was represented by a single bead positioned at the Cα atom. The system was maintained at a simulation temperature of 0.4 reduced units (which corresponds to about 300 K) (Bigman and Levy 2021) and an ionic strength of 0.02 M. The apiCCT domains were modeled by a native‐topology‐based Lennard‐Jones potential (extracted from the 7LUP crystal structure) to reward native contacts and penalize non‐native interactions, Debye‐Hückel potential (Azia and Levy 2009) to account for long‐range electrostatic interactions, and a hydrophobicity scale (Dannenhoffer‐Lafage and Best 2021) to explicitly capture residue‐specific hydrophobic interactions. The positively charged residues (Lys, Arg) were assigned a point charge of (+1*e*) and the negatively charged residues (Asp, Glu) were assigned a negative charge of (−1*e*). The tau^4R^ protein was modeled as an intrinsically disordered protein without any native structure bias, allowing for a wide range of movement in its bond angles and dihedral angles, consistent with its natural flexibility and conformational heterogeneity. The dynamics were simulated using the Langevin equation. Simulations were performed within a cubic box measuring 250 × 250 × 250 Å, thereby providing sufficient spatial freedom. Each system underwent 10 independent simulations, each consisting of 10^8^ steps with a time step of 2 fs, resulting in a total simulation time of 2 μs per system. The interaction probabilities between tau^4R^ and apiCCT residues are presented using heat maps, aggregated from the coarse‐grained molecular dynamics simulations. Pairwise distances between residues of tau^4R^ and apiCCT were calculated throughout the simulations, with an interaction defined as occurring when the distance between residue centers was less than 6 Å, which is appropriate for a CG C_α_‐based model. Interaction data were binned into groups of three consecutive residues for both tau^4R^ and apiCCT. PyMOL3 software was used to construct the initial protein configurations and visualize surface charges.

## AUTHOR CONTRIBUTIONS

**Miki Ben‐Maimon:** Investigation; writing – original draft; formal analysis. **Nadav Elad:** Investigation; writing – review and editing. **Segev Naveh‐Tassa:** Investigation; writing – review and editing. **Yaakov Levy:** Funding acquisition; supervision; writing – review and editing. **Amnon Horovitz:** Conceptualization; funding acquisition; writing – original draft; formal analysis; supervision.

## Supporting information


**Figure S1.** Reproducibility of tau^4R^ aggregation curves. The kinetics of tau^4R^ (10 μM) fibril formation were monitored by measuring ThT fluorescence, as described in section 3. Shown are 20 independent repetitions demonstrating the reproducibility of the aggregation kinetics.
**Figure S2.** Nano differential scanning fluorimetry of the CCT3, CCT7, and CCT8 apical domains. The proteins were diluted to 25 μM in the final solution used for the aggregation assays (39 mM HEPES (pH 7.4) 39 mM KCl, 60 mM NaCl and 2.4 mM Tris–HCl (pH 8.0)). Analysis was performed using the NanoTemper Prometheus NT.Plex device by measuring fluorescence at 330 and 350 nm. The first derivative of the 330/350 nm fluorescence ratio is plotted as a function of temperature. Data represent two replicates per apical domain. Melting curves characteristic of well‐folded domains were observed for apiCCT3, apiCCT7, and apiCCT8.
**Figure S3.** Effects of the CCT3 and CCT7 apical domains on the kinetics of tau^4R^ aggregation. The data in Figure 1 for different concentrations of apiCCT3 (blue) and apiCCT7 (red) were fitted to Equation (1). Shown are the half‐life times and rate constants obtained from those fits. The data points for tau^4R^ alone are represented by red dots in blue circles. Standard errors were less than 5%.
**Figure S4.** Distinguishing between aggregation mechanisms. Plots of ThT fluorescence as a function of time at varying tau^4R^ concentrations either alone (a) or in the presence of 2.5 μM apiCCT7 (b) were subjected to global fitting using AmyloFit (Meisl et al. 2016). Given that the double‐logarithmic plots of aggregation half‐time versus monomer concentration are found to be linear in both cases (Figure 3), only mechanisms for which no curvature is expected were considered. The lowest mean squared residual errors (MRE), 0.0215 for tau^4R^ alone and 0.0097 in the presence of apiCCT7, are found for the fragmentation model, thereby indicating that it is the most likely mechanism. In the case of the nucleation elongation and secondary nucleation models, the respective MRE values are 0.0714 and 0.0716 for tau^4R^ alone and 0.0362 and 0.0386 when apiCCT7 is present.

## Data Availability

The data that support the findings of this study are available from the corresponding author upon reasonable request.
