# Supplementary material for: Inhibition of tau aggregation by the CCT3 and CCT7 apical domains
Source: Protein Sci. 2025 May 22;34(6):e70162. doi: 10.1002/pro.70162 (PMC12095920; doi:10.1002/pro.70162)
Supplement: Supplementary file 1 — Figure S1. Reproducibility of tau4R aggregation curves. The kinetics of tau4R (10 μM) fibril formation were monitored by measuring ThT fluorescence, as described in section 3. Shown are 20 independent repetitions demonstrating the reproducibility of the aggregation kinetics. Figure S2. Nano differential scanning fluorimetry of the CCT3, CCT7, and CCT8 apical domains. The proteins were diluted to 25 μM in the final solution used for the aggregation assays (39 mM HEPES (pH 7.4) 39 mM KCl, 60 mM NaCl and 2.4 mM Tris–HCl (pH 8.0)). Analysis was performed using the NanoTemper Prometheus NT.Plex device by measuring fluorescence at 330 and 350 nm. The first derivative of the 330/350 nm fluorescence ratio is plotted as a function of temperature. Data represent two replicates per apical domain. Melting curves characteristic of well‐folded domains were observed for apiCCT3, apiCCT7, and apiCCT8. Figure S3. Effects of the CCT3 and CCT7 apical domains on the kinetics of tau4R aggregation. The data in Figure 1 for different concentrations of apiCCT3 (blue) and apiCCT7 (red) were fitted to Equation (1). Shown are the half‐life times and rate constants obtained from those fits. The data points for tau4R alone are represented by red dots in blue circles. Standard errors were less than 5%. Figure S4. Distinguishing between aggregation mechanisms. Plots of ThT fluorescence as a function of time at varying tau4R concentrations either alone (a) or in the presence of 2.5 μM apiCCT7 (b) were subjected to global fitting using AmyloFit (Meisl et al. 2016). Given that the double‐logarithmic plots of aggregation half‐time versus monomer concentration are found to be linear in both cases (Figure 3), only mechanisms for which no curvature is expected were considered. The lowest mean squared residual errors (MRE), 0.0215 for tau4R alone and 0.0097 in the presence of apiCCT7, are found for the fragmentation model, thereby indicating that it is the most likely mechanism. In the case of the nuclea [file PRO-34-e70162-s001.pdf]

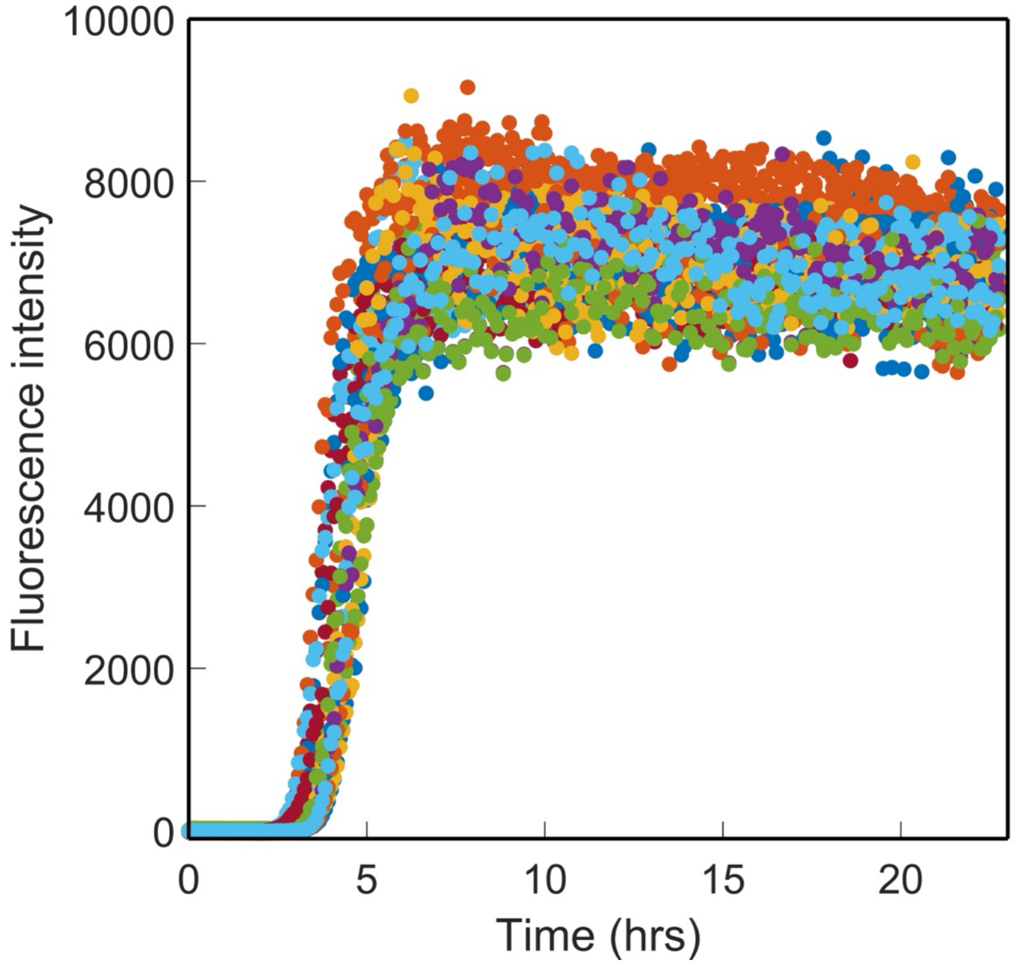

Figure S1. Reproducibility of tau[4R] aggregation curves. The kinetics of tau[4R] (10 μM) fibril formation were monitored by measuring ThT fluorescence, as described in the Materials and Methods. Shown are 20 independent repetitions demonstrating the reproducibility of the aggregation kinetics.

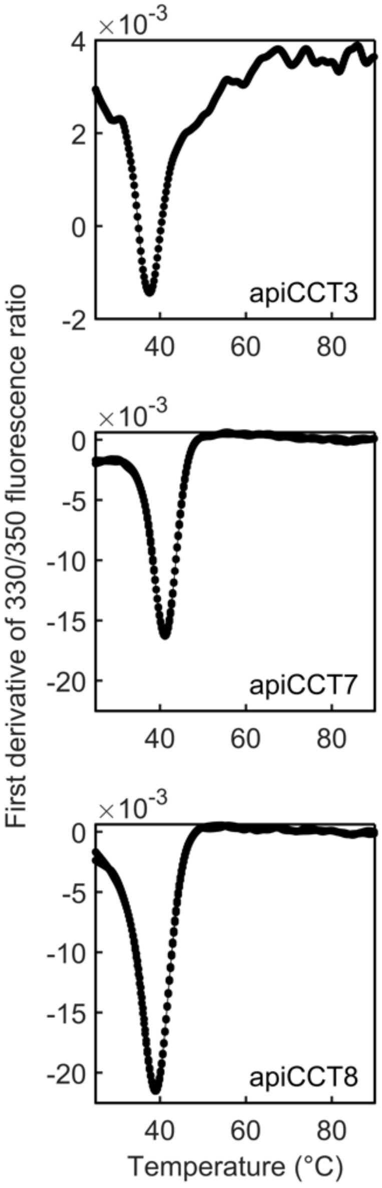

Figure S2. Nano differential scanning fluorimetry of the CCT3, CCT7, and CCT8 apical domains. The proteins were diluted to 25 µM in the final solution used for the aggregation assays (39 mM HEPES (pH 7.4) 39 mM KCl, 60 mM NaCl and 2.4 mM Tris-HCl (pH 8.0)). Analysis was performed using the NanoTemper Prometheus NT.Plex device by measuring fluorescence at 330 and 350 nm. The first derivative of the 330/350 nm fluorescence ratio is plotted as a function of temperature. Data represent two replicates per apical domain. Melting curves characteristic of well-folded domains were observed for apiCCT3, apiCCT7 and apiCCT8.

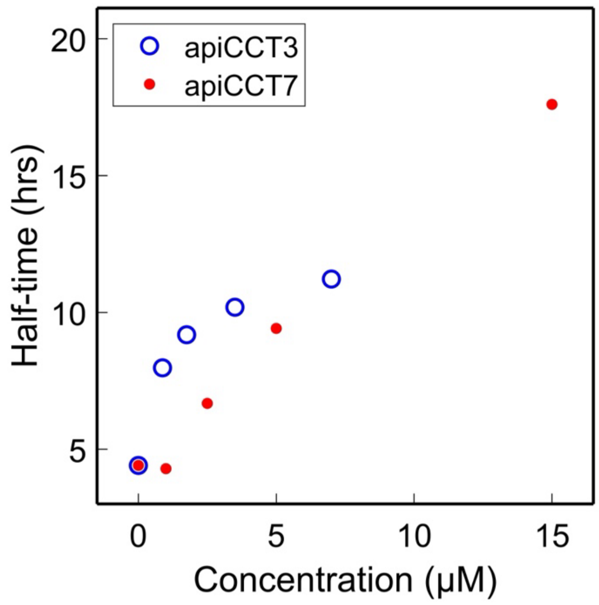

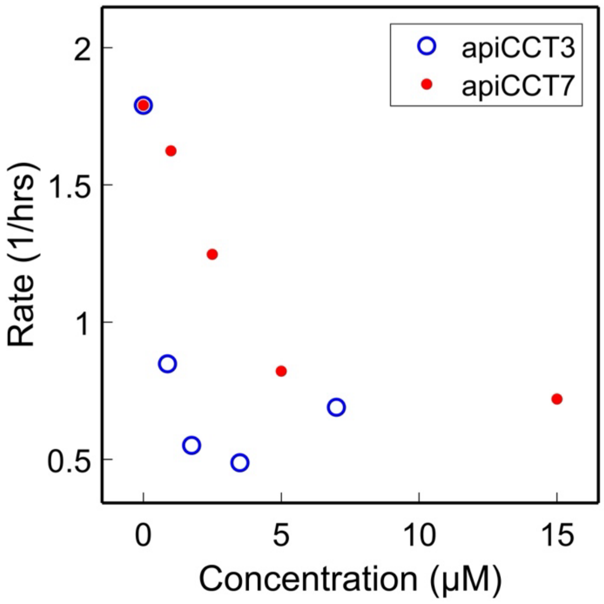

Figure S3. Effects of the CCT3 and CCT7 apical domains on the kinetics of tau^{4R} aggregation. The data in Figure 1 for different concentrations of apiCCT3 (blue) and apiCCT7 (red) were fitted to Equation (1). Shown are the half-life times and rate constants obtained from those fits. The data points for tau^{4R} alone are represented by red dots in blue circles. Standard errors were less than 5%.

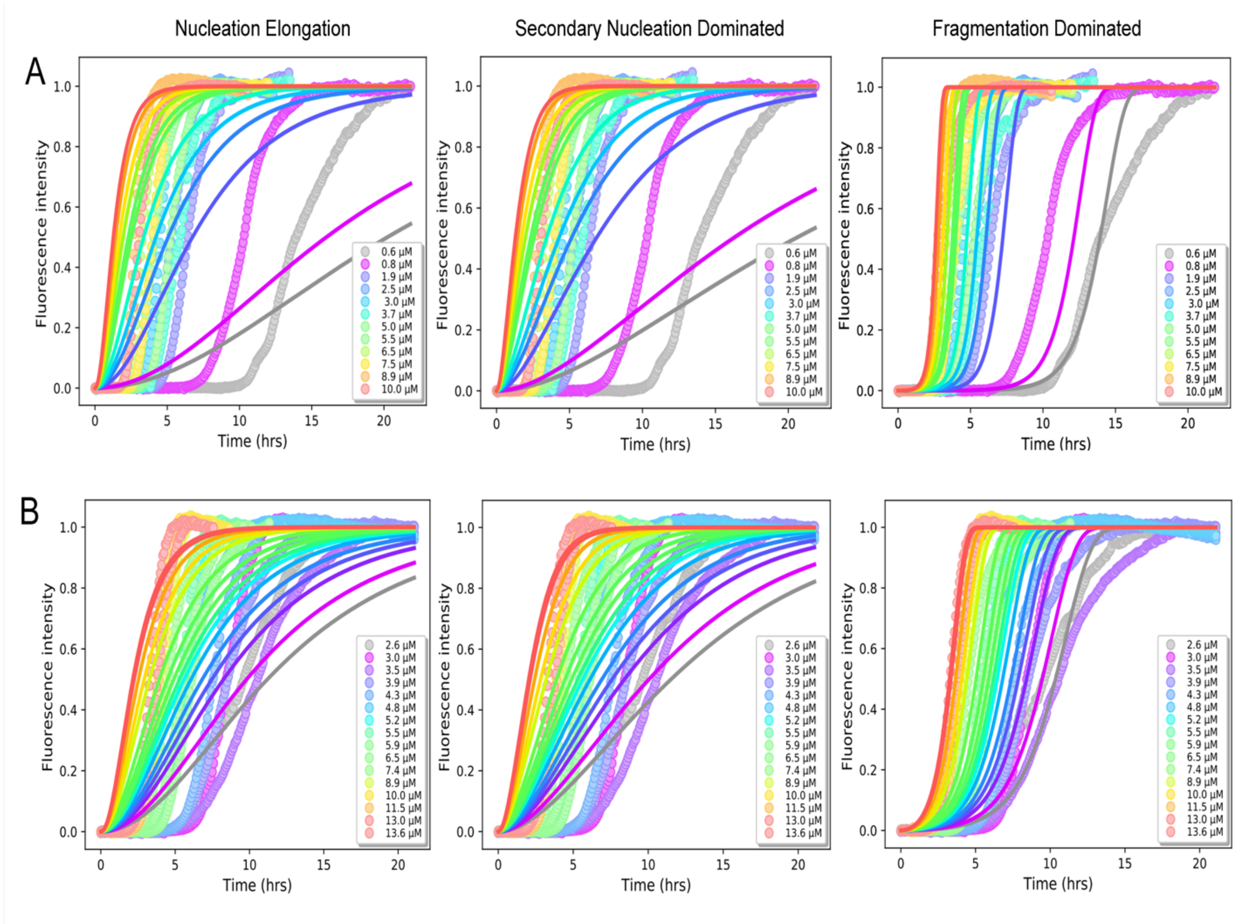

Figure S4. Distinguishing between aggregation mechanisms. Plots of ThT fluorescence as a function of time at varying tau^{4R} concentrations either alone (A) or in the presence of 2.5 µM apiCCT7 (B) were subjected to global fitting using AmyloFit (Meisl et al., 2016). Given that the double-logarithmic plots of aggregation half-time vs. monomer concentration are found to be linear in both cases (Figure 3), only mechanisms for which no curvature is expected were considered. The lowest mean squared residual errors (MRE), 0.0215 for tau^{4R} alone and 0.0097 in the presence of apiCCT7, are found for the fragmentation model, thereby indicating that it is the most likely mechanism. In the case of the nucleation elongation and secondary nucleation models, the respective MRE values are 0.0714 and 0.0716 for tau^{4R} alone and 0.0362 and 0.0386 when apiCCT7 is present.
